# The Book World of Medicine and Science

**Published:** 1911-04-15

**Authors:** 


### The Book World of Medicine and Science.

### MASSAGE.

Text-book of Massage. By L. L. Despard. (London: Henry Frowde and Hodder and Stoughton. Oxford University Press, Warwick Square, E.C. 1911. Price 10s. 6d.)

We took up this work with a lively sense of satisfaction to find that here, at last, was an authoritative work on massage. Our first impressions, we confess, were entirely favourable. The excellent anatomical preface, illustrated by the accurate and artistic blocks from Cunningham, proved wholly excellent, and the introductory chapter on massage in general so comprehensive, concise, and clear, and withal so orthodox, that we eagerly anticipated an equally excellent second part. Unfortunately, however, the second part of the book, excluding that section which is devoted to a description of electrical apparatus (which is on the whole good) hardly comes up to the promise held out by the first.

It is a common error that there exists an "English method" of massaging which is different from that of the Continental schools. As a matter of fact, the differences between the art of English masseurs and their (in general) far more skilled Continental confrères is a difference of degree, a difference of application of certain general principles. We hold no brief for the Continental system, but we merely state a well-known fact when we say that there is in this country no school of massage that can compete with the excellent institutions that exist in Sweden, in Germany, and in France. There a medical masseur—as distinct from a mere "rubber" who plies his art at the baths—has to undergo a long and arduous training, extending over eighteen months at least and, if electrical work is included, over three years. With us there are "schools" which turn out so-called masseurs after three months' teaching! The deduction is obvious, and need not be further commented upon. But it is worth while to point out that this haphazard manner of dealing with what is after all a highly skilled profession re-acts to the disadvantage of the medical profession, the masseurs, and the general public. If we had in this country capable masseurs such as are available on the Continent, where a medical man can send a patient with a written card to any trained masseur and rely upon his instructions being carried out with the necessary skill, the requisite anatomical knowledge on the part of the masseur, and the thoroughness which massage demands to be effective, we should hear far less than we do at present about the wonders of bone-setters, of vibratory treatment, and similar popular lines. Since we do not possess such skilled masseurs—or rather since their number is so few that they may be counted on the fingers, the public has begun to lose faith in ordinary medical massage and has patronised the advertising quacks who, to do them credit, have really, in many cases, taken pains to become competent masseurs and medical gymnastic experts. It is a well-known fact that there are at the present moment several such "outside firms" in London that are treating patients sent to them by medical men, and in the circumstances no one can blame the profession for taking advantage of their existence to obtain from them benefits which it cannot obtain from recognised masseurs.

A glance at Miss Despard's book will show, to those who understand the art of massage, the importance of the points to which we refer. Here we have an authoritative work on the art of massage, a book which one would naturally expect to be the best English text-book on the subject. Yet many of the descriptions are inaccurate, and many of the important details are slurred over. To start with the first movement; Miss Despard writes, "Pressure should be made in one direction only, the hand or fingers at the end of each stroke being passed lightly backwards over the surface of the skin to the starting-point." This is certainly not effleurage, whatever it might be. What she probably means is that the stroking must be made in one direction only, but she fails to add that that direction is a very definite one, the direction in fact of the lymphatics leading towards the nearest set of glands. In the forearm, it is now generally conceded, the first gland lies some distance below the bend of the elbow; the condylar gland comes next, and yet in the figure accompanying the text, the hands of the operator are shown stroking the limb downwards towards the wrist joint. In the limbs, of course, this detail is not of great importance, but in the massage of individual muscles—for instance the trapezius—it is of primary importance. Nor is requisite stress laid on the position of the hands in making this effleurage movement—which the author holds to be equivalent to a simple process of stroking. The proper position is to have the hands in close apposition to the muscles, but in no single case is this shown in the illustrations. Fig. 122 is not an illustration of effleurage. Nor do we understand what Miss Despard means when she writes about "friction." Her description is simply that of a superficial movement, whereas the true friction movement is one in which the skin is kept almost perfectly steady while the tissues underneath it are massaged—a movement which, we admit, is very difficult to learn and still more difficult to describe correctly in print. So too in petrissage—which by the way is not merely kneading—the skin is never lifted up, as Miss Despard says when describing "superficial petrissage." Probably here again she has something else in mind—the "rolling movement" which at one time was much used by the German school—but that is quite a different thing from petrissage and must not be confounded with it. Nor is her description of the "deep-kneading" movements easy to comprehend. The essential movement in petrissage starts at the origin of a muscle, not at its insertion—that is to say, at its fixed point and away from the nearest set of glands—a little thing, but it is little things that make for perfection, while to employ a well-worn adage, perfection itself is not a little thing. It is just by attention to these little details that the masseur secures perfection. The illustration on page 189 showing circumduction of the arm is a very curious one. The patient's deltoid is bunched up and the forearm flexors are in full action, while the operator is apparently compressing the subclavian from behind. Equally wrong, in our opinion, is the method of circumducting the thigh shown in fig. 140.

In exercising certain sets of muscles it is important to bear in mind that all the muscles must be relaxed except that group for which the exercise is being made. The sole exception to this rule is the resistive exercise in which the synergic muscles are in full action. But Miss Despard seems to disregard this rule, and after carefully perusing her instructions in cases of deformity and scoliosis, one is left in doubt as to what these exercises really are. With regard to abdominal massage, we notice that more than half a page is given to massage of the liver, and that Miss Despard recommends both effleurage and petrissage for this organ. While we do not deny that these movements are excellent for the abdominal musculature, we utterly fail to conceive what possible effect they can have on a viscus which is protected for the most part by the ribs. The essential massage of the liver is tapotement. Again, under the heading "Chorea" she recommends effleurage and deep petrissage as the best movement: with this opinion we do not fancy most medical men will agree.

An important omission in Miss Despard's book is all detail with regard to examining patients, and hints as to the observation of certain conditions which the masseur should always bear in mind. To take one instance, in her otherwise excellent description of the movements in writers' cramp, she altogether omits to warn the operator of the possibility of a neuritis supervening. When neuritis complicates a case of graphospasm it is obvious that the ordinary movements in massage must be omitted: probably Miss Despard would reply that it is the medical man's business to note the onset of neuritis, but it is equally much the masseurs business to be on the look out for it. The recommendations with regard to bandages, lotions, electrical treatment and other points are very good and are given with commendable clearness and force.

We have drawn attention to certain defects, as they appear to us, in this otherwise valuable work, not from a spirit of mere cavilling but because we think it important that a work such as this, which is eminently worthy of being considered a good text-book on an important subject should be faultless on points of technique. Doubtless the reply to our objections would be that the method herein described is the English or rather the American method. Dr. Kellog's book is frequently referred to, but Dr. Arvedson's work is not even mentioned. A manual of massage should take some notice of what has been done in the art by various other workers in the field if it wishes to be considered an authoritative work on the subject with which it deals. This book ignores so much that it cannot honestly lay claim to that honour.

### THERAPEUTICS.

"Salvarsan" or "606" (Dioxydiamidoarsenobenzol). Its Chemistry, Pharmacy, and Therapeutics. By W. Harrison Martindale, Ph.D. Marburg, F.C.S., and W. Wynn Westcott, M.B. Lond., D.P.H., H.M.'s Coroner for North-East London. (London: H. K. Lewis. 1911. Price 5s. net.)

Numerous have been the papers written about this so-called specific for syphilis, and it is almost an impossibility for the general practitioner to find time to study from them the question of salvarsan, even were he brave enough to attempt the task. The appearance of this work will therefore be welcomed by such a one, for in it he will find information digested and analysed under certain headings in a manner that cannot fail to be of assistance to him. The interest which the lay press has taken in this much-advertised remedy make it absolutely necessary for every physician to have a working knowledge of the drug, for the public has come to consider it a panacea for syphilitic lesions in all their stages, as well as an instantaneous cure for the disease when recently acquired. It is well, therefore, that the physician should be in a position to educate his patient on the subject should the drug be mentioned by the latter. Much disappointment may in this way be avoided, for arsenic is still on its trial, at any rate as far as this country is concerned, in the treatment of spirochætal diseases. The work before us opens with a chapter on the chemistry of "606," and after the chapter on its chemistry come others on its therapeutic uses, its results in the various stages and types of syphilis, in parasyphilitic diseases, in spirilosis in general, and in syphilis complicated by other diseases, such as phthisis. Next come chapters on dosage, methods of injection, and descriptions of apparatus necessary for its administration. Then a full account is given of untoward results experienced with the drug and of contraindications to its use. The volume is brought to a close by a number of special communications on the use of salvarsan contributed by well-known English authorities, and by a full bibliography of original papers arranged in chronological order from March 15 to December 17, 1910. It is impossible to do otherwise than congratulate the authors on the adequate and impartial manner in which they have stated the case for "606."

### MISCELLANEOUS.

Aids to Midwives. By Rebecca Emily Rémy, L.O.S., C.M.B. (London: The Scientific Press. Pp. 34. 1911. Price 1s.)

We may suppose that Miss Rémy would be the last to admit that in writing for midwives it is unnecessary to trouble about one's English, but in this pamphlet we find errors even in the first paragraph, and the prejudice thus early awakened is not allayed by proceeding further. No doubt many of the mistakes are but slips, though that does not absolve the authoress from the charge of gross carelessness in preparing the matter for the press. In writing for such a class as midwives it behoves an author to pay particular attention to accuracy and simplicity, and not to assume that readers will be able to distinguish slips of the pen. There is, of course, contained in the pages a certain amount of correct instruction, but this hardly justifies the production of this book, seeing that there are already excellent text-books to be had. Many of the procedures mentioned are beyond the scope of such a book, and if they were not, the descriptions given are too limited to be of much practical assistance to a midwife. Miss Rémy has failed to take the best advantage of the sources from which she has obtained the information she has thought advisable to retail in this fashion; while the unfortunate inaccuracies and deplorable English leave us in no mood to point out *seriatim* the individual defects, even if we had the space to do so.

Gynæcology. "Catechism Series." (Edinburgh: E. and S. Livingstone. 1911. Pp. 84 + iv. Price 1s. net).

This new addition to the well-known "Catechism Series" of Messrs. Livingstone is issued anonymously. It makes no pretensions to being a text-book, but is merely a means of rapidly reviewing knowledge obtained from other sources. Provided that the student bears this fact in mind he may make use of the volume with benefit, for the information it gives is accurate as far as it goes, though necessarily somewhat sketchy.

Legends of Our Lord. By Mrs. Arthur Bell. (London: Kegan Paul, Trench, Trübner & Co.)

As is to be expected from these publishers, the many illustrations contained in this book are equally well chosen and produced. As to the text, that is rather an arbitrary selection from the host of legends which continued to grow around the figure of Christ till Renaissance days. Now, many of these legends present Him in a very human fashion. For instance, those which abound in the Apochryphal Gospels, and these Mrs. Bell has omitted, on the ground no doubt that modern people would regard them as in the worst taste. As the book is popular and not scholastic in aim no one will quarrel with Mrs. Bell for this, and hospital secretaries and matrons who wish to add to their library something that will be read with delight in the children's ward, are recommended to buy this volume without delay. At Easter particularly it is likely to be appreciated.

